# Bacteriophage Therapy to Control Bovine Mastitis: A Review

**DOI:** 10.3390/antibiotics12081307

**Published:** 2023-08-10

**Authors:** Janet Y. Nale, Neil R. McEwan

**Affiliations:** 1 Centre for Epidemiology and Planetary Health, Scotland’s Rural College (SRUC), Inverness IV2 5NA, Scotland, UK; 2 School of Veterinary Medicine, Scotland’s Rural College (SRUC), Aberdeen AB21 9YA, Scotland, UK; neil.mcewan@sruc.ac.uk

**Keywords:** *Staphylococcus*, *Streptococcus*, *Enterococcus*, *Actinomyces*, *Aerococcus*, *Escherichia*, *Klebsiella*, *Proteus*

## Abstract

Bovine mastitis is a polymicrobial disease characterised by inflammation of the udders of dairy and beef cattle. The infection has huge implications to health and welfare of animals, impacting milk and beef production and costing up to EUR 32 billion annually to the dairy industry, globally. Bacterial communities associated with the disease include representative species from *Staphylococcus*, *Streptococcus*, *Enterococcus*, *Actinomyces*, *Aerococcus*, *Escherichia*, *Klebsiella* and *Proteus*. Conventional treatment relies on antibiotics, but antimicrobial resistance, declining antibiotic innovations and biofilm production negatively impact therapeutic efficacy. Bacteriophages (phages) are viruses which effectively target and lyse bacteria with extreme specificity and can be a valuable supplement or replacement to antibiotics for bovine mastitis. In this review, we provide an overview of the etiology of bovine mastitis, the advantages of phage therapy over chemical antibiotics for the strains and research work conducted in the area in various model systems to support phage deployment in the dairy industry. We emphasise work on phage isolation procedures from samples obtained from mastitic and non-mastitic sources, characterisation and efficacy testing of single and multiple phages as standalone treatments or adjuncts to probiotics in various in vitro, ex vivo and in vivo bovine mastitis infection models. Furthermore, we highlight the areas where improvements can be made with focus on phage cocktail optimisation, formulation, and genetic engineering to improve delivery, stability, efficacy, and safety in cattle. Phage therapy is becoming more attractive in clinical medicine and agriculture and thus, could mitigate the impending catastrophe of antimicrobial resistance in the dairy sector.

## 1. Impact of Bovine Mastitis

Mastitis is a condition which manifests as inflammation of the tissue of the mammary gland. Specific to this review, which deals with it in the context of dairy cattle, it is regarded as one of the major sources of potentially avoidable economic loss within the dairy sector. Although it has been described across a wide range of mammalian species, it has generally been most widely studied either in lactating dairy cattle or in women who are breastfeeding. For example, in humans, it has been estimated that as many as one in five women who are breastfeeding are affected by mastitis [1], generally within the first couple of months after giving birth. However, although most of the scientific literature deals with cases in cattle and humans, there are lots of examples of it occurring in other species [2]. 

Bovine mastitis is normally regarded as more of an issue in dairy cattle, most probably because at a clinical level it is seen manifesting as swelling and/or a general redness of the udder which is easily visible during times of milking on the farm. However, mastitis can also be a problem in beef cattle, albeit often at a subclinical level where there is no obvious visible sign of the condition, but there can still be some level of impact on the health of the animal. For example, in one study, it was noted that around half of the beef cattle studied had signs of subclinical mastitis, based on somatic cell counts collected from milk from these animals [3]. While this adversely affected the cow, it did not impact on the weaning weight of the calf she was nursing.

In the beef cow, providing the calf being suckled is supplemented with sufficient milk for its growth, the financial implications of a mastitis infection may not have an appreciable effect and so would go unnoticed as part of normal animal husbandry. However, it is easier to detect and assess the impact in the dairy herd, where a cow is not left to feed her calf postpartum, and mastitis can be seen at the time of milking. In 2015, the financial costs of any mastitis infection in the first 30 days of a lactation were estimated at USD 444 over the course of the lactation which followed [4]. Of this, around 30% of the figure was estimated to be due to direct costs such as treatment and immediate reduction in milk production, with the remainder including factors such as longer-term loss of milk later in the lactation. However, this figure is for a single cow that develops mastitis. Across the global dairy sector, it was estimated in 2016 that the financial impact of mastitis is anywhere from EUR 19.7 billion to EUR 32 billion (https://www.thecattlesite.com/articles/mastitis-where-are-we-at-now, accessed on 17 July 2023).

## 2. Microorganisms Causing or Associated with Bovine Mastitis

Although the main cause of mastitis is bacterial sources, this is not because of any one particular species as there are a number of different species which can cause the condition. One estimate had the range of possible species which are capable of being involved in mastitis sitting at as many as thirty-six different species or sub-species [5]. Thirty-four of these were isolated in a single investigation of twenty herds of cattle, where every herd was affected to some extent, with a range of between four and twenty-one different species from any given herd.

The major organisms (represented by >1% of all Isolates) identified in the work of Aarestrup et al. were generally members of the genus *Staphylococcus*, most commonly *S. aureus*, *S. chromogenes*, *S. epidermidis*, *S. haemolyticus*, *S. simulans*, and *S. warneri*, together with organisms from other genera, namely *Streptococcus dysgalactiae*, *S. uberis*, *S. canis* and *Enterococcus faecalis* [5].

However, the researchers also succeeded in isolating other members of the genus *Staphylococcus* at lower frequencies, namely *S. auricularis*, *S. capitis*, *S. cohnii*, *S. hominis*, *S. lentis*, *S. muscae*, *S. saprophyticus* and *S. sciuru*, together with non-specified/unidentified members of the same genus. The other sources of infection which they isolated were *Actinomyces pyogenes*, *Aerococcus hydro*, *A. viridans*, *E. avium*, *E. durans*, *E. faecium*, *Escherichia coli*, *Klebsiella pneumoniae*, unidentified members of the genus *Proteus*, *S. lactis* and *S. salivarius*. However, this is just an example of a single case study, and other studies have reported cases where some of the organisms found at minor levels were either present at higher relative abundance or as the sole or predominant causal organism, or indeed found other organisms capable of causing mastitis [6].

An example of another organism with the potential to cause mastitis is *S. agalactiae*. This is an organism which has been isolated from a range of different animals, including non-mammalian species [7], demonstrating that its role goes beyond causing mastitis. The example of *S. agalactiae* is an interesting one as it is not only able to infect a range of host species, but it can also be the causal organism for more than one clinical condition. For example, as well as being an organism which can lead to bovine mastitis, it has been described as being responsible for both sepsis and meningitis in humans, and also meningoencephalitis in fish [7]. Moreover, even at subclinical levels, *S. agalactiae* has been shown to have a detrimental impact on milk production, in terms of both quantity and milk quality [8]. Although a fairly large number of species are included in the list above, there are other examples of species which have been associated with the onset of mastitis. These include *K. oxytoca* [9], *Mycobacterium bovis* [10], *Pseudomonas aeruginosa* [11] and *S. xylosus* [12,13].

In the context of the spread of infectious agents, it has been shown that the transmission of strains of *S. aureus* found on human skin, or at least strains present as part of the endogenous population on the hands of those milking the cows, are unlikely to be responsible for the introduction of mastitis into a herd of cattle [14]. In addition, this work also demonstrated that there was relatively little variation in the major strains causing acute clinical mastitis on a single farm, with around 90% of these strains being the same. In addition, these strains were generally more difficult to eradicate following treatment. However, in terms of inter-farm comparisons, it was observed that there were differences in terms of the predominant strains on the different farms. This suggests that although human-to-cow transmission is not high, the transmission of strains from one cow to another cow in the same herd is high, particularly in the case of more problematic strains [14]. The exact cause of this remains unknown as a number of potential avenues for transmission are possible, e.g., from the milking machine, from any cloths used to wipe udders, or even from strains which are transiently present on the hands of milking staff. Although the microbial community of the human hands is regarded as ordinarily unlikely to be a source of new mastitis infections, they are considered to be potentially capable of transmitting infections from one infected animal to another.

It has been predicted that the list of species known to be associated with mastitis will grow with the application of next-generation sequencing becoming a more commonly used research tool. By use of metagenomic sequencing, the relative abundance of various different organisms was seen to differ between cows which were healthy and those showing signs of clinical mastitis [15]. In addition to changes in bacterial species, there were also examples of archaeal and viral species only detected in the cattle with clinical mastitis [15]. However, it remains unclear how many of the organisms detected by metagenomic approaches are causal agents for mastitis and which ones are secondary or opportunistic colonisers following other organisms causing the development of mastitis conditions.

## 3. Treatment for Mastitis and Potential Problems with Current Control Methods

Traditionally, the most common method to treat cases of mastitis has been the use of antibiotics, although there are several examples of strains of certain species where antibiotic resistance has been described for isolates obtained from mastitis animals. Examples can be seen in penicillin resistance in *S. aureus* [6,16] and *S. epidermidis* [5] together with both tetracycline resistance and streptomycin resistance in *S. epidermidis* [5]. In addition to the spread of antibiotic resistance genes, the potential effects of antibiotics can be reduced by protection afforded to bacteria found within biofilms. This includes examples of strains of organisms associated with mastitis, that were isolated from milk, and evidence was obtained to show that *S. aureus* biofilms are more likely to be associated with intramammary infections than the teats [17,18].

The obvious multidrug resistance shown towards a wide range of chemical antimicrobials are compelling and triggers the urgent need to identify and develop alternative strategies to control bovine mastitis safely and effectively [19,20]. The risks and effects of bacterial resistance are not restricted to cattle herds but possibly to humans via contaminated products such as milk and beef [21,22]. There is also a huge potential for environmental contamination and spread to different niches [23]. Several strategies which focus on diagnostic, therapeutic and managemental approaches to target and control bovine mastitis have been identified and have clear potential to replace or supplement antibiotics [24]. Therapeutic strategies such as the use of antimicrobial peptides, probiotics, herbal therapy, immunotherapy, nanoparticle-based approaches, stem cells and native secretory factors have strong prospects in their own merits to control the disease in cattle [24]. These strategies have also been shown to have better outcomes if combined with other managemental practices such as genetic selection, nutritional changes, dry cow and lactation therapy, the use of teat sealant to prevent contamination arising from the environment and acoustic pulse therapy [25].

A novel and emerging treatment which explores the application of bacteriophages (phages, viruses of bacteria) has been shown to greatly mitigate bacterial resistance and can improve the general health and production capacity of livestock [20,26]. Pertinent to bovine mastitis, several research studies which were dedicated to the isolation, characterisation, and safety and efficacy testing of therapeutic phages in appropriate model systems have been reported. Therefore, here, in the subsequent section of this review, we provide a detailed overview of such work conducted in the area and the various steps taken to ascertain the therapeutic potential of bovine mastitis phages for clinical deployment in the cattle industry. Subsequently, we suggest ways in which the phages can be improved through careful selection of strictly lytic or virulent phages, genetic engineering to improve lysis efficiency, phage cocktail optimisation to target the polymicrobial niche of bovine mastitis and to mitigate phage resistance, and formulation strategies to enhance phage stability, delivery and efficacy in cattle. More detailed information on the above bacteria which have phages described for them in the context of bovine mastitis is presented in Table 1, but at this point the extensive list has been included here with a view to illustrating the range of organisms which are known to either cause or at least have been identified as being associated with bovine mastitis.

## 4. The Case for Phage Therapy to Control Bovine Mastitis

Phages specifically infect bacteria with a resultant outcome of either a lysis/killing of the bacterial host (lytic or virulent phages) or a lysogeny—the integration of phage genetic material into the host bacterial chromosome (temperate phages) [70]. Several phage characteristics offer attractive mechanisms to enable their therapeutic deployment to effectively control multidrug-resistant bacteria in veterinary medicine including treatment of bovine mastitis as discussed below [26,71].

### 4.1. Phage Specificity, Lysis and Amplification

The phage target specificity means that they cause minimal disruption to the normal microbiomes of animals, thus preserving the beneficial microbial niche [72]. The precise bacterial selection by the phage is achieved by recognising specific receptor proteins on the host bacterium which the phage adsorb to using specialised tail fibers; after which they penetrate and release their genetic material into the host [73]. Generally, phages of most *S. aureus* strains interact with a unique cell wall teichoic acid, which is different from other coagulase-negative staphylococci (CoNS) and blocks recognition by phages specific to CoNS using the tail tip complex [74]. For studies specifically conducted on bovine mastitis, *S. aureus* phages utilise three domains located on the endolysin sequences: cysteine; histidine-dependent amidohydrolase/peptidase (CHAP); amidase 2 (N-acetylmuramyl-L-alanine amidase); and SH3b for host cell wall recognition [42].

Sequel to successful adsorption and penetration, lytic phages immediately hijack their host DNA replication machinery to synthetise genetic materials and structural proteins during the latent period. The time taken to achieve this has been reported to vary in bovine mastitis phages and could range from 10 (*S. aureus)*, 15 (*E. faecum*), 20 (*P. aeruginosa* phage), to 30 min (*S. agalactiae*) [7,50,67,69]. Subsequently, after viral synthesis, numerous phage particles are assembled and eventually released by the lysis of the host through a combined activity of the endolysin and holin enzymes that degrade the bacteria cell wall [75]. For bovine mastitis, phage progeny or burst (number of phage particles synthesised and released per single bacterial cell) varies from 20 to 100 PFU/cell within ~175 min [7,27,50,67,69]. The ability of lytic phages to ultimately lyse infected bacteria and amplify after infection ensures the clearance of the bacterial pathogens as well as continual increased supply of infective phages (auto-dosing) at infection sites [26,71]. Furthermore, the shorter replication time demonstrated by the phages can reduce product development timeframes to provide an opportunity for rapid customised or tailored treatments to target specific strains of bacteria [26].

**Figure 1:**
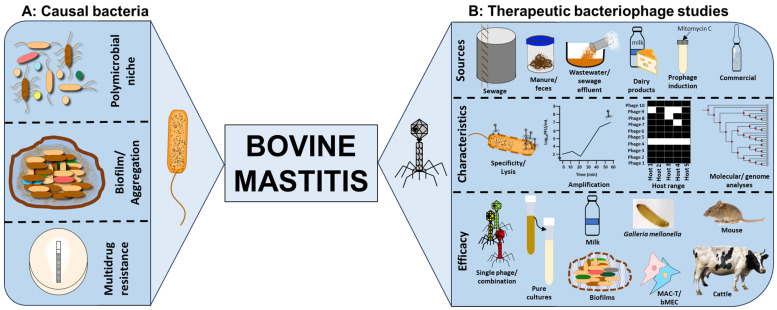
Characteristics of bacterial communities that cause bovine mastitis and aspects of bacteriophage therapeutic studies conducted in the area to control the infection. (**A**) The microbial niche of bovine mastitis consists of polybacterial strains which can aggregate and produce biofilms and are mostly resistant to several antimicrobials which affect antibiotic efficiency. (**B**) Phages that target and kill the bacterial species have been isolated either directly or via enrichment of samples from sewage, wastewater and sewage effluent, manure and faeces of cattle and pigs, and dairy products such as cheeses and raw or unpasteurised milk, via prophage induction of bacterial cultures with mitomycin C or commercially sourced. Phage characterisation focuses on determining lysis specificity and efficacy, phage infection kinetics to ascertain the adsorption, replication/amplification and growth, host range coverage (black cells showing bacterial lysis by phages and white cells showing no lysis), and genome analyses to ascertain gene functions, expressions and relationships. Therapeutic activities of single and combinations of phages were tested in various infection models in vitro (bacterial clearance in pure cultures, milk and biofilms), ex vivo (in cell cultures of MAC-T and bMEC) and in refined (*Galleria mellonella* larvae) and established (mice and cattle) in vivo models. The mouse and cattle images were downloaded from Microsoft PowerPoint resources under the license CC BY-NC and no modifications were made.

For bovine mastitis, there is also a range in the types of phages which have been identified as being candidates for treatment of one or more of the organisms known to be an infectious agent. Examples of the bacterial species which are known to be involved in causing mastitis, together with the types of phages which can infect them, and the outcomes of the conducted work are listed in Table 1 and Figure 1. Several phages have been identified for these purposes, but a greater proportion of work has been conducted on *S. aureus* being the common aetiological agent causing this infection. This approach has also been described for bacterial species such as *A. viridans* [68], *E. coli* [60] and *K. oxytoca* [9].

### 4.2. Isolation of Phages from a Wide Range of Sources

Phages are the most abundant entities on earth with a ~10^31^ PFU/mL reported concentration [76,77,78]. For bovine mastitis, various sample sources have been explored for the purpose of isolating phages for the various pathogens responsible for the infection (Table 1). Most of the work has focused on screening raw milk samples obtained from a confirmed mastitis cattle, either directly after centrifuging and filtering of samples or via enrichment procedures to amplify and isolate phages [28,34]. Phages have been reported to actively bind to, lyse and amplify in milk constituents, and huge successes of phage isolation for *S. aureus*, *S. agalactiae* and *S. arlettae* have been recorded from this source through this method [7,28,29,34,40,41,46,49,63]. However, in one instance, no phage was isolated from the milk samples examined [48]. The reason for this may be attributed to the bovine whey protein which may prevent attachment of some phages [58]. This may also simply be the lack of phages specific for the bacterial host used as a target for the isolation or the occurrence of the phages in very low titers requiring an enrichment procedure to enable viral amplification and enhance detection [48].

Milk products have also been examined and yielded phages for bovine mastitis pathogens *Staphylococcus* and *Streptococcus* via enrichment of Cabrales and Peñamellera cheeses, although all phages isolated from this method yielded temperate phages [34]. Moreover, *S. aureus* and *S. arlettae* phages of lysogenic origin have been isolated from milk as well [34,40,41,56,63] (Table 1), although for regulatory purposes, lytic phages are preferred to temperate phages due to the possibility of lysogeny occurring and transfer of virulence genes via horizontal gene transfer. However, where strictly lytic phages are not isolated, temperate phages showed potential therapeutic efficacy and are particularly useful for treatment [79,80].

Sewage, sewage effluent, sewage water, barn flushes, wastewater, cowshed water and manure from dairy farms have yielded a large quantity of phages targeting mastitis-causing pathogens which may be attributed to the microbial richness in these sources [32,37,42,43,48,51,52,53]. Other very odd sources such as pig manure have been a good source to isolate phages for the infection; this may reveal the interconnection of niches for these organisms [50,54,55] (Table 1).

Phages from commercial sources such as 23361 (ATCC), BP39 (PhageLux) and SAML-4, SAML-12, SAML-150, SAML-4229 and SATA-8505 (StaphLyse) have been investigated for potential usage for bovine mastitis *S. aureus* [30,31].

### 4.3. Cocktail Optimisation to Improve Therapeutic Activity

Therapeutic activity of single-phage treatments can significantly reduce bacterial load in many infection models using optimal multiplicity of infection (MOI; the ratio of infecting phages to bacteria in a given infection challenge) as shown in many studies [32,43,48,49,51,52,53,60]. However, phage resistance was detected within as early as 2 h after phage treatment as indicated by the regrowth of cultures after lysis which can negatively impact therapeutic efficacy [49]. To curtail resistance and lysogeny development, broaden host target coverage and specificity, and to improve lysis efficiency, a cocktail of diverse phages can be optimised [62,81,82,83]. This strategy has proven successful, and various combinations of diverse phage morphologies have shown beneficial combinatorial effects in clearing several bacteria causing bovine mastitis. For example, a four-phage cocktail was developed for *E. coli* and cocktails of two or three phages were shown to be more effective than single-phage treatments for *S. aureus* [30,34,35,54,55,61,62]. Similarly, the therapeutic efficacy of a phage cocktail was shown to be comparable to that of the antibiotic ceftiofur sodium for *E. coli* in cattle and *S. aureus* in mice [35,61]. This has been extrapolated further, with a cocktail of four phages together with the lactic acid bacterium *Lactiplantibacillus plantarum* proving effective [54]. Phage activity on *S. aureus* was shown to be delayed by *IgG*-dependent aggregation using single-phage treatment. While in contrast, the use of a cocktail showed no significant effect with or without *IgG* in milk [30].

### 4.4. Characterisation of Phage Lysis and Stability in Pure Cultures

Several therapeutic assessments have investigated the efficacy and safety of phages for the targeted eradication of bovine mastitis. Fundamental research has been conducted regarding phage activity in pure cultures to determine lysis capabilities by individual phages and in combination with other phages. Host range analysis mainly focuses on phage lysis activity using spot test with the double-layer agar method (application of phage samples to confluent cultures of bacteria in a semi-solid agar medium overlayed on solid agar medium). This is to ascertain the range of relevant bacterial strains the phages can lyse with some demonstrating broad or narrower host coverage [28,36,37] (Table 1). Besides phage coverage on a wide range of strains, other phages of *S. aureus* showed inter-species lysis, targeting *S. sciuri* and *Rothia terrae* [28] as well as *E. coli* [43], and *K. oxytoca* phage P2 lysing *E. aerogenes* as well [9]. 

Further work was also directed to stability (in various temperature and pH ranges) and killing assays in pure cultures in broth or liquid media and milk (pasteurised and unpasteurised) using MOI assays in a given infection model to provide an insight into the dosage [54,55]. Data showed a wide range of effectiveness of MOI range of 0.001 to 100 in vitro [43,48,49,51,52,53,63,68]. However, optimal effectiveness was at MOI of 10 in vivo for some of the data [60]. Other reports showed that efficacy was achieved in a phage-dose-dependent manner in milk using an *S. aureus* phage [31].

### 4.5. Phage Therapeutic Activity in Biofilms

The pathogens causing mastitis can aggregate in vitro and in vivo in extracellular polysaccharide-containing biofilm matrixes which restricts antibiotic access to bacteria [17,18,47,84]. Phages have been shown to prevent or penetrate established biofilms produced by mastitis bacteria in vitro and in vivo, hence showing the potential to be used as a standalone treatment or to supplement antibiotic use and enhance therapeutic efficacy [47,49]. The phages can lyse bacteria early in the culture to prevent biofilm formation or may disrupt established biofilms which can enhance bacterial killing or provide pathogens access within the biofilm matrix [72,82]. In *S. aureus* biofilms, treatment using a single phage or a cocktail of phages significantly reduced bacterial load in planktonic cultures as well as established biofilms on polystyrene surfaces, in milk and on mammary glands [47,49].

As well as the issue of potential protection from biofilms, mastitis-causing bacteria have been shown to be afforded some level of protection from bacterial aggregation [56], including during the times when *S. aureus* was exposed to phage infection. However, previous work showed a total kill of *S. aureus*, which has a few cells and have survived phage treatment, probably by some level of aggregation. This means that the numbers remaining are sufficiently low for them in turn to be removed by the animal’s own immune system [30].

### 4.6. Phage Therapeutic Assessments in Mastitis Ex Vivo and In Vivo Models

Phages have low inherent toxicity to the immune system, and they are potentially cheaper to isolate and develop, which provides an economic advantage over antibiotics [72]. To contextualise and provide insight into the therapeutic safety and efficacy of phages, relevant ex vivo models involving bovine cells lines were investigated. The studied cell lines for bacterial and phage interactions for this are the mammary alveolar cells-large T antigen (MAC-T) and bovine mammary epithelial (bMEC) cell lines [51,52,65]. A cocktail of two phages, CM8-1 and SJT-2 was shown to reduce *K. pneumonia* numbers and consequently reduce adhesion, invasion, and cytotoxicity in bMEC cells [65]. *S. aureus* phages were shown to migrate intercellularly and could reach the nucleus within 3 h after exposure to MAC-T cell lines and have an endocytotic activity of 12% in a bovine ex vivo model [51,52].

Studies on *S. aureus*-colonised *G. mellonella* larvae showed a 50% survival rate four days after treatment with a single phage [44]. The in vivo model that has been extensively studied for bovine mastitis phage therapy is the mouse model mainly because this model has itself been well established for infection since the 1970s [35,44,50,60,64,68]. Results in mice showed favourable outcomes for phage therapy with reduced colonisation and reduced inflammatory cytokines as soon as 24 h after treatment. The mouse model has also been reported to be a more time- and cost-effective model than those of larger mammals with comparable symptoms, inflammatory indicators, colonisation, and histopathological characteristics. Therapeutic efficacies have been achieved in cattle as well [57,61].

## 5. Barriers/Challenges to Therapeutic Phage Application to Control Bovine Mastitis

We outlined the advantages of phage therapy and research work conducted in the area to control bovine mastitis. However, a degree of caution needs to be applied by anyone considering using it as a potential prophylactic treatment. It has been reported that the infusion of a phage sample into unaffected quarters in the udder of lactating dairy cattle resulted in an increase in the somatic cell count in the milk from that quarter [57]. This suggests that there has been some form of immune response taking place in that particular quarter of the cow’s udder. A comparable increase in somatic cell count was not seen in animals infused with a phage sample where the animal had some level of mastitis infection, even at a sub-clinical level [57].

The situation in terms of using phage as a treatment for mastitis is complicated, yet evidence exists to show that in *S. aureus* the whey proteins in milk can adhere to the surface of cells, thereby blocking potential attachment sites for the phage [58]. Moreover, it was shown that in raw milk, as opposed to milk which has been heat-treated, phage K which has the potential to infect and kill *S. aureus* was less successful [59]. It is thought that this is due to the clumping of the bacteria on fat globules within the milk and some sort of presumed protection from this activity.

On the other hand, the lysogenisation of the bacterial host by temperate phages could potentially cause the exchange of virulence factors via horizontal gene transfer as stated above. However, the use of phages can come with additional complications. One such example of this was seen where a phage which entered the lysogenic phase was also found to contain a gene which conferred resistance to multiple types of antibiotics [12]. Therefore, although there is a clear potential for usage of phages as a means of killing bacteria causing mastitis infections, there needs to be considerable research undertaken before using these phages as treatments. Temperate phages can access the lytic lifecycle via induction through treatment with mitomycin C as shown in *S. galactiae* [56] or activation of the repressor or deletion of the integrase genes. Unfortunately, they are unsuitable for therapeutic purposes in their wild form. However, genetic engineering has provided avenues for genetic manipulation to help develop therapeutically acceptable phages where strictly lytic ones are not available [85,86,87].

The polymicrobial niche of bovine mastitis is also a challenge to overcome [5,6,16]. Most work conducted to date has focused on a single bacterial species in relevant infection model systems, except for example where *S. aureus* phages showed interspecies lysis on *S. sciuri* and *Rothia terrae* [28], and *E. coli* [43]. Whilst this is informative and provides useful insights into the therapeutic potential of the phages, it is still unclear how these single bacterial species targets would alleviate bovine mastitis. More work is therefore needed on multispecies targets through phage cocktail optimisations to clear the bacterial communities as standalone treatments or as adjunct to antibiotics for the effective clearance of bovine mastitis infection.

## 6. Thoughts on Phage Purification and Formulation for Safe and Optimal Delivery

The majority of phages targeting bovine mastitis are stable in a wide temperature and pH ranges, are effective in various infection models and have the potential to migrate within and between mammalian cells and lyse bacteria [51,52]. However, it is unclear whether the results obtained from the controlled laboratory assays can be directly extrapolated to application in the intended animal species and whether all phages would maintain their efficacy when applied in clinical settings.

Also, the phages tested in the various models are produced under experimental laboratory conditions and in the bacterial medium which may contain high levels of endotoxins such as the lipopolysaccharides of Gram-negative bacteria. Previous in vivo studies purified phage lysates using Cesium chloride to remove endotoxins [61]. Generally, endotoxins can be removed from phage preparations by treatment with polyethylene glycol, ultrafiltration, gel filtration, anion-exchange chromatography, octanol extraction, deoxycholate extraction or endotoxin removal columns, and these methods have been extensively discussed previously [88]. These methods can be explored to produce purified phage lysates for the use in bovine mastitis.

Phages are composed of proteins and nucleic acids, and can be highly unstable once removed from their bacterial medium, exposed to adverse conditions or in clinical settings [89]. Hence, there is the need for the phages to maintain viability or shelf life in storage to ensure they are optimally delivered to infection sites and can maintain therapeutic efficacy in these conditions. The different methods by which phages can be stabilised to enhance activity and delivery have been clearly described in a recent review [90]. Phages can be formulated using encapsulation strategies through emulsification, freeze drying, spray drying, liposomal encapsulation, entrapment and electrospinning to ensure optimal delivery and stability [90]. Furthermore, phage immobilisation methods such as through physical, charge-directed, protein–ligand and covalent immobilisation strategies have been shown to help stabilise phages and improve shelf life and binding efficiency [90]. The processes for achieving each method, their advantages in phage therapy and ways to mitigate or improve the strategies for tested phages were described. As expected, all processes would depend on the property of the phage in question and convenience and ease of application of the formulation in the context of bovine mastitis.

## 7. Conclusions

MDR is a huge problem to the health and welfare of livestock, and in particular, it is a threat to the dairy industry. Conventional treatment of bovine mastitis relies on antibiotics, but MDR that are reported in the bacterial etiological agents pose a huge challenge to animals, humans, and the environment. Phages infect and kill bacteria with great specificity and here, we reviewed therapeutic work conducted in the targeted eradication of the bacteria responsible for bovine mastitis, focusing on methods of sample collection and phage isolation procedures and characterisation in various infection models. We also emphasised difficulty involving the selection of strictly lytic phages to optimise cocktails to target bacterial communities and potential ways in which the phages can be developed to enhance therapeutic activity. The clear advancements made in phage therapeutic studies discussed here show great prospects for bovine mastitis and pave the way for clinical deployment in the very near future, with the hope that appropriate phages will be isolated and tested within the next decade. This review contributes immensely to the control of bovine mastitis and global AMR crises in the livestock industry.

## Figures and Tables

**Table 1:** Summary of all potential therapeutic phages identified for the treatment of bovine mastitis.

Bacteria		Phages		Therapeutic Activity	
Species	Resistance	Name (Morphology/Classification *)	Source	Outcome	Reference(s)
*Staphylococcus aureus*	MDR	SAH-1 (M)	Enrichment of sewage effluent	Latent period of 20 min and burst of 100 PFU/cell, significantly reduced bacterial growth at MOIs of 1–100	[27]
		B_UFSM4^L^ (S) B_UFSM5^L^ (S)	Coagulase-positive Staphylococcus bovine milk	Broad host range (B_UFSM4, 45.8%; B_UFSM5, 4.16%; n = 24), intra-species infection on *S. sciuri* and *Rothia terrae*	[28]
	MRSA	Six, only three fully characterised	Milk	Reduced *S. aureus* CFU counts by 64–95%	[29]
		ATCC 23361 (M) BP39 (R)	In-house directed evolution (ATCC 23361) Commercial (PhageLux, BP39)	Phage cocktail was effective in milk in vitro with/without supplementation with *IgG.* Reduced colonisation, high intramammary phage counts recorded, no phage systemic spread in mouse model	[30]
	MSSA, MRSA VISA	SAML-4 (H) SAML-12 (H) SAML-150 (H) SAML-4229 (H) SATA-8505 (H)	Commercial (StaphLyse^TM^)	Wide host range (92.7% at 10^4^ CFU/mL and 100% at 10^9^ CFU/mL of 709 strains). Phages were stable at 4 °C and 37 °C and activity was dose dependent in milk. Reduced colonisation in mouse mammary gland 8 h after treatment and prophylactically 4 h before challenge was most effective	[31]
	Penicillin Ampicillin	Ufv-aur2 (M) Ufv-aur3 (M) Ufv-aur4 (M) Ufv-aur5 (M) Ufv-aur6 (M) Ufv-aur7 (M) Ufv-aur8 (M) Ufv-aur9 (M) Ufv-aur10 (M) Ufv-aur11 (M)	Sewage water	Reduced bacterial growth after 8 h, thermostable between 70 °C and 100 °C, lysed 80–100% of 20 isolates examined	[32]
	Ampicillin	vB_SauM-UFV_DC4 (M)	Wastewater of dairy farm	UFV_DC4 lysed two of the strains examined	[33]
		C1^L^ ** P1^L^ ** L7^L^ ** L13^L^ ** A8^L^ ** H5^L^ (S) A72^L^ (S)	Enrichment of Cabrales cheese, Peñamellera cheese and raw milk	H5 and A72 were characterised, stable at 0–4 °C but reduced by 20–30% at 22–37 °C, respectively. Also stable at 72 °C for 15 s but inactivated after 1 min. Challenge assay in vitro showed bacterial inhibition in UHT and pasteurised milk but reduced activity in semi-skimmed and whole raw milk	[34]
		vBSM-A1 (M) vBSP-A2 (P)	Mixed sewage samples from cattle farms	A cocktail of two phages was superior to single-phage treatments and comparable to ceftiofur sodium in mice; it improved mastitis pathology and reduced colonisation. High intramammary phage recovery was observed without systemic spread	[35]
	Cefoxitin, Oxacillin, Vancomycin	SA (M)	Wastewater	Stable at pH 4–11 and temperatures 28–37 °C but significantly reduced at 50–105 °C. Host range of 50% (n = 12). Significantly reduced bacterial growth 8 h a phage treatment	[36]
	MRSA	PhiSA012 (M) PhiSA039 (M)	Previously isolated from sewage influent	PhiSA012 and 039 showed variable host range. SA012 activity was delayed by bovine *IgG* dependent aggregation. Intravenous and intra-peritoneal administration of SA012 reduced bacterial colonisation in and inflammation of mammary gland	[37,38,39]
		JS01^L^ (S)	Milk of mastitis cows	43,458 bp genome of 66 ORFs, 33.32%, G/C content and no tRNAs. Encodes two virulence factors, staphylokinase and Staphylococcal complement inhibitor	[40,41]
		Phage 1 ** Phage 2 ** Phage 3 ** Phage 4 ** Phage 5 **	Barn flush water from four dairy herds	Phage 2 and 4 showed wide host range lysing 69–100% susceptibility (n = 100). Highly conserved endolysin with 99% similarity to other Staphylococcal phages. Three domains for phage involved in phage recognition and bacterial lysis were identified	[42]
		*PSW* (M)	Wastewater from dairy farm	65–69 kb genome, small burst of 44 ± 3 PFU/mL/bacteria, attachment not influenced by calcium, stable at 40–60 °C and pH 2–9, resistant to chloroform, optimal lysis MOI is 0.01. Inhibited growth of four *S. aureus* strains and showed interspecies infection on *E. coli*	[43]
	MRSA MSSA	Romulus (T) Remus (T) ISP (T) DSM105264 (Phage K, K)	Sewage water (Romulus and Remus), Unknown sources (ISP, Phage K)	Romulus, Remus, ISP showed lysis activity. A 50% survival in *Galleria mellonella* 4 days after phage treatment and incomplete recovery in mice 48 h after phage treatment with ISP phage	[44]
		ΦMSP (S)	Sewage	Possessed hydrolase of 70 kDA and induced twenty-six *S. aureus* proteins during infection	[45]
	MDR	Phage 3 ** Phage 7 ** Phage 8 ** Phage 15 ** Phage 17 ** Phage 18 ** Phage 19 **	Milk from mastitis cows shedding Staphylococci	Phages lysed both bovine and human bacterial isolates; they have similar plaque morphology to phages from human sources, not stable beyond 67 °C. No significant difference in susceptibility to mercuric chloride, hydrogen ion concentrations, sterile water or saline. Sterile water was toxic to low-concentrated phages	[46]
	MRSA	vB_SauM_SDQ (M)	Sewage	Lysed 20 of 24 strains, reduced established biofilms on polystyrene, milk, and mammary gland tissue after treatment	[47]
	MRSA MSSA	Phage 24 A2 **	Cowshed wastewater	Lysed 19 of 30 strains examined. Phage cleared bacterial cultures on agar at MOI of 10, supporting topical application for therapeutic use	[48]
	MDR	4086-1 (P) 4086-2 (P) 4086-3 (P) 4086-4 (P) 4086-6 (P)	Milk samples from mastitis cows	Phages 4086-1, 4086-2 and 4086-3 lysed four, while 4086-4 and -6 lysed two of the six *S. aureus* strains tested. Significantly reduced bacterial load at MOI 0.1, 2–4 h after phage treatment in vitro but resistance was observed 2–5 h afterwards. Significantly reduced biofilm mass and colonisation in the mammary gland, decreased expression of TNF-α and IL-6, reduction in mammary infiltration of *S. aureus* in mouse model	[49]
	MRSA	SLPW (P)	Faecal sewage in a pig farm	Lysed 36 of 40 isolates examined. Stable at up to 45 °C, chloroform and ultraviolet light but deactivated at 65 °C. Short latent (10 min), long lytic period (120 min), intraperitoneal phage administration remedially reduced colonisation and inflammation of cytokines in mice, effective in intra-abdominal infection for different MLST types	[50]
		vB_SauM_JS25 (M)	Sewage effluent in a dairy farm	Lysed 51 of 56 strains tested, stable at pH 6–9, deactivated at 70–80 °C for 10 min, significantly reduced bacterial load at MOI 1 in vitro. Ex vivo assays using MAC-T showed phage reached nucleus 3 h after infection and reduced colonisation in a time-dependent manner intracellularly; endocytotic activity was at 12%	[51,52]
		vB_SauS_IMEP5 (S)	Manure from dairy farms	Stable at pH 3–10, inactivated at 70 °C for 20 min, reduced bacterial growth at MOI 0.001	[53]
		TA1.ST29 (M) EB1.ST11 (P) EB1.ST27 (P)	Sewage water (TA1.ST29) Pig manure (EB1.ST11 and EB1.ST27)	Two of three of bacterial isolates were lysed by at least a single phage, cocktail of the three phages along with and in combination with *L. planetarium* significantly reduced colonisation in pasteurised and raw milk	[54,55]
*Streptococcus agalactiae*		LYGO9^L^ (S) HZ04^L^ (S) pA11^L^ (S)	Induction with mitomycin C	Specificity to *S. galactiae*; lysed 12, 13, 20 of 42 strains examined	[56]
		Bacteriophage K (K)	Unknown	Whey protein in milk could inhibit phage adhesion and proliferation in milk. Intramammary infusion of phages reduced colonisation in 16.7% of treated lactating cows. Large increases in somatic cells were observed in phage-treated healthy cows	[57,58,59]
		JX01^L^ (S)	Milk of mastitis cows	~90% of phage adsorbed after 2.5 min, burst of 20/cell, latent period of 30 min. Deactivated at 60 °C at 30 min, with ~70% reduction at 50 °C.	[7]
*Escherichia coli*	MPEC	vB_EcoM_UFV13 (T)	Sewage	Stable at pH 4–12, temperature 37–62 °C, activity optimal at 22–37 °C and not affected by osmotic shock and organic solvents Sarksoyl and CTAB. A 10-fold reduction in bacterial load was observed at MOI of 10 in mice. From seven pro-inflammatory cytokines (IL-6, TNF-α, IL-2, IFN-γ, IL-4, IL-10 and IL-17A,) only IL-10, IL-6 and TNF-α expressions were statistically significant	[60]
	MDR	vB_EcoM_SYGD1 (M) vB_EcoP_SYGE1 (A) SYGMH1 (M)	Sewage of dairy farms	Stable at 25–37 °C, deactivated at 60 °C. Optimal pH range is 5–9 and sensitive to ultraviolet light. Cocktail of the phages reduced colonisation, somatic cells, and inflammatory factors, alleviated symptoms of mastitis in cattle. Results were comparable to ceftiofur sodium-treated group	[61]
	Ampicillin	Four-phage cocktail	Sewage wastewater	Significant reduction in bacterial counts in raw milk and adherence to bovine mammary alveolar epithelial cell line, MAC-T	[62]
*Klebsiella oxytoca*		P1 (M) P2 (M) P3 (P) P4 (M)	Wastewater	Stable at 37–50 °C, inactivated at pH 2, 5 and 11. Treatment caused 97% reduction in bacterial growth in pure cultures. P2 showed interspecies lysis clearing *Enterobacter aerogenes* as well.	[9]
*Staphylococcus arlettae*		BM31^L^ (S)	Milk of bovine mastitis cows	Stable at pH 6–9, temperatures 40–50 °C but significantly reduced at 60 °C, and in chloroform and ether. Optimal MOI was 0.001 and 1. First phage to be isolated for this bacterium	[63]
*Klebsiella pneumoniae*	MDR and non-MDR	M_Kpn_HB132952 (S) CM_Kpn_HB143742 (P)	Sewage samples	Optimal MOI is 0.01 for M_Kpn_HB132952 and 1 for CM_Kpn_HB143742, pH 4–11, and 30–60 °C. Both phages had similar host range (30/31 strains), TNF-α and IL-1β expression not significantly different between treated and untreated mice	[64]
		CM8-1 *** SJT-2 ***	Dairy farm wastewater	Phage treatment reduced bacteria adhesion, invasion and cytotoxicity. Phage treatment suppressed morphological changes in bMECs 4–8 h after treatment. Phage treatment mitigated expression of TLR4, NF-κB, TNF-α, IL-1β, IL-6, IL-8, caspase-3, caspase-9 and cyt-c in bMECs and increased apoptosis of bMECs	[65]
		CM8-1 ***	Dairy farm wastewater	Stable at 30–50 °C, pH 6–10, reduced colonisation 2 h after phage treatment in mammary glands, reduced expression of TNF-α, IL-1β, IL-6, and IL-8 in murine model	[66]
*Pseudomonas aeruginosa*	MDR	vB_PaeS_PAJD-1 (S)	Sewage from dairy farm	Short latent period of 20 min, stable at 25–55 °C and pH 5–9. In murine model, phage treatment significantly reduced colonisation and repaired mammary glands	[67]
*Aerococcus viridans*		vB_AviM_AVP **	Sewage	Optimal MOI was 0.001. Stable at pH 3–11, 25–50 °C. Reduced colonisation in damaged breast of mice with no bacteria detection with 10^7^ PFU of phage treatment for 24 h. No significant difference in CFU load was recorded for 10^5^ PFU treatment compared to control treatment with PBS. Reduced TNF-α, IL-1β, and IL-6 expression, and myeloperoxidase activity	[68]
*Enterococcus faecium*		vB_EfaM_XJ3 (M)	Dairy cattle faecal sample	Optimal MOI was 0.001, latent period was 15 min, burst 84 and burst time was 175 min, stable at 50 °C and pH 5–11	[69]

* Published classification/morphology as revealed in the articles. These are: S (*Siphoviridae*), M (*Myoviridae*), H (*Herelleviridae*), T (*Tevenvirinae*), A (*Autographiviridae*), D (*Drexlerviridae*), R (*Rountreeviridae*) (*Kayvirus* of the subfamily *Spounavirinae).* ** No morphology or classification were provided in the publications. *** Ultra-structures of phages were described but not the classification. ^L^ Lysogenic/temperate phages isolated either through prophage induction or from enrichment of samples. Table was constructed from combined outputs from searches conducted on Web of Science, PubMed and Google Scholar. Work was restricted to whole phages only on organisms examined in the context of bovine mastitis. Abbreviations used in this table are: ISP (intravenous staphylococcal phage); MDR (multidrug resistance); MOI (multiplicity of infection); MPEC (mammary pathogenic *Escherichia coli*); MRSA (methicillin-resistant *Staphylococcus aureus*); MSSA (methicillin-sensitive *Staphylococcus aureus*); ORF (open reading frame); PFU (plaque forming unit); VISA (vancomycin intermediate *Staphylococcus aureus*), bMEC (bovine mammary epithelial cells), MAC-T (Mammary Alveolar Cells—large T antigen cells).

## References

[1] Bond D.M., Morris J.M., Nassar N. (2017). Study protocol: Evaluation of the probiotic Lactobacillus Fermentum CECT5716 for the prevention of mastitis in breastfeeding women: A randomised controlled trial. BMC Pregnancy Childbirth.

[2] Radostits O.M., Gay C.C., Hinchcliff K.W., Constable P.D. (2007). Veterinary Medicine: A Textbook of the Diseases of Cattle, Horses, Sheep, Pigs and Goats.

[3] Persson Waller K., Persson Y., Nyman A.K., Stengärde L. (2014). Udder health in beef cows and its association with calf growth. Acta Vet. Scand..

[4] Rollin E., Dhuyvetter K.C., Overton M.W. (2015). The cost of clinical mastitis in the first 30 days of lactation: An economic modeling tool. Prev. Vet. Med..

[5] Aarestrup F.Μ., Wegener H.C., Rosdahl V.T., Jensen Ν.E. (1995). Staphylococcal and other Bacterial Species Associated with Intramammary Infections in Danish Dairy Herds. Acta Vet. Scand..

[6] Aarestrup F.M., Wegener H.C., Rosdahl V.T. (1995). A comparative study of *Staphylococcus aureus* strains isolated from bovine subclinical mastitis during 1952–1956 and 1992. Acta Vet. Scand..

[7] Bai Q., Zhang W., Yang Y., Tang F., Nguyen X., Liu G., Lu C. (2013). Characterization and genome sequencing of a novel bacteriophage infecting *Streptococcus agalactiae* with high similarity to a phage from *Streptococcus pyogenes*. Arch. Virol..

[8] Elias A.O., Cortez A., Brandão P.E., da Silva R.C., Langoni H. (2012). Molecular detection of *Streptococcus agalactiae* in bovine raw milk samples obtained directly from bulk tanks. Res. Vet. Sci..

[9] Fahliyani S.A., Beheshti-Maal K., Ghandehari F. (2018). Novel lytic bacteriophages of *Klebsiella oxytoca* ABG-IAUF-1 as the potential agents for mastitis phage therapy. Fems Microbiol. Lett..

[10] Farzaneh M., Derakhshandeh A., Al-Farha A.A.A., Petrovski K., Hemmatzadeh F. (2022). A novel phage-displayed MilA ELISA for detection of antibodies against Myc. bovis in bovine milk. J. Appl. Microbiol..

[11] Giannattasio-Ferraz S., Ene A., Gomes V.J., Queiroz C.O., Maskeri L., Oliveira A.P., Putonti C., Barbosa-Stancioli E.F. (2022). *Escherichia coli* and Pseudomonas aeruginosa Isolated From Urine of Healthy Bovine Have Potential as Emerging Human and Bovine Pathogens. Front. Microbiol..

[12] Wipf J.R., Schwendener S., Perreten V. (2014). The novel macrolide-Lincosamide-Streptogramin B resistance gene erm(44) is associated with a prophage in Staphylococcus xylosus. Antimicrob. Agents Chemother..

[13] Richards V.P., Zadoks R.N., Pavinski Bitar P.D., Lefébure T., Lang P., Werner B., Tikofsky L., Moroni P., Stanhope M.J. (2012). Genome characterization and population genetic structure of the zoonotic pathogen, *Streptococcus canis*. BMC Microbiol..

[14] Larsen H.D., Sloth K.H., Elsberg C., Enevoldsen C., Pedersen L.H., Eriksen N.H., Aarestrup F.M., Jensen N.E. (2000). The dynamics of *Staphylococcus aureus* intramammary infection in nine Danish dairy herds. Vet. Microbiol..

[15] Hoque M.N., Istiaq A., Clement R.A., Sultana M., Crandall K.A., Siddiki A.Z., Hossain M.A. (2019). Metagenomic deep sequencing reveals association of microbiome signature with functional biases in bovine mastitis. Sci. Rep..

[16] Aarestrup F.M., Wegener H.C., Rosdahl V.T. (1995). Evaluation of phenotypic and genotypic methods for epidemiological typing of *Staphylococcus aureus* isolates from bovine mastitis in Denmark. Vet. Microbiol..

[17] Bissong M.E.A., Ateba C.N. (2020). Genotypic and Phenotypic Evaluation of Biofilm Production and Antimicrobial Resistance in *Staphylococcus aureus* Isolated from Milk, North West Province, South Africa. Antibiotics.

[18] Fox L.K., Zadoks R.N., Gaskins C.T. (2005). Biofilm production by *Staphylococcus aureus* associated with intramammary infection. Vet. Microbiol..

[19] Li X., Xu C., Liang B., Kastelic J.P., Han B., Tong X., Gao J. (2023). Alternatives to antibiotics for treatment of mastitis in dairy cows. Front. Vet. Sci..

[20] Cheng W.N., Han S.G. (2020). Bovine mastitis: Risk factors, therapeutic strategies, and alternative treatments—A review. Asian-Australas. J. Anim. Sci..

[21] Pomba C., Rantala M., Greko C., Baptiste K.E., Catry B., van Duijkeren E., Mateus A., Moreno M.A., Pyörälä S., Ružauskas M. (2016). Public health risk of antimicrobial resistance transfer from companion animals. J. Antimicrob. Chemother..

[22] Saini V., McClure J.T., Léger D., Keefe G.P., Scholl D.T., Morck D.W., Barkema H.W. (2012). Antimicrobial resistance profiles of common mastitis pathogens on Canadian dairy farms. J. Dairy Sci..

[23] Skandalis N., Maeusli M., Papafotis D., Miller S., Lee B., Theologidis I., Luna B. (2021). Environmental Spread of Antibiotic Resistance. Antibiotics.

[24] Sharun K., Dhama K., Tiwari R., Gugjoo M.B., Iqbal Yatoo M., Patel S.K., Pathak M., Karthik K., Khurana S.K., Singh R. (2021). Advances in therapeutic and managemental approaches of bovine mastitis: A comprehensive review. Vet. Q..

[25] El-Sayed A., Kamel M. (2021). Bovine mastitis prevention and control in the post-antibiotic era. Trop. Anim. Health Prod..

[26] Makumi A., Mhone A.L., Odaba J., Guantai L., Svitek N. (2021). Phages for Africa: The Potential Benefit and Challenges of Phage Therapy for the Livestock Sector in Sub-Saharan Africa. Antibiotics.

[27] Han J.E., Kim J.H., Hwang S.Y., Choresca C.H., Shin S.P., Jun J.W., Chai J.Y., Park Y.H., Park S.C. (2013). Isolation and characterization of a Myoviridae bacteriophage against *Staphylococcus aureus* isolated from dairy cows with mastitis. Res. Vet. Sci..

[28] Barasuol B.M., Cargnelutti J.F., Sangioni L.A., Pereira D.I.B., Varela A.P.M., Mayer F.Q., Pottker E.S., Goncalves G.F., Cibulski S., Botton S.D. (2022). Characterization of novel of temperate phages of *Staphylococcus aureus* isolated from bovine milk. Arch. Microbiol..

[29] Basdew I.H., Laing M.D. (2015). Investigation of the lytic ability of South African bacteriophages specific for *Staphylococcus aureus*, associated with bovine mastitis. Biocontrol Sci. Technol..

[30] Breyne K., Honaker R.W., Hobbs Z., Richter M., Żaczek M., Spangler T., Steenbrugge J., Lu R., Kinkhabwala A., Marchon B. (2017). Efficacy and safety of a bovine-associated *Staphylococcus aureus* phage cocktail in a murine model of mastitis. Front. Microbiol..

[31] Brouillette E., Millette G., Chamberland S., Roy J.P., Ster C., Kiros T., Hickey S., Hittle L., Woolston J., Malouin F. (2023). Effective Treatment of *Staphylococcus aureus* Intramammary Infection in a Murine Model Using the Bacteriophage Cocktail StaphLyse™. Viruses.

[32] Dias R.S., Eller M.R., Duarte V.S., Pereira Â.L., Silva C.C., Mantovani H.C., Oliveira L.L., Silva Ede A., De Paula S.O. (2013). Use of phages against antibiotic-resistant *Staphylococcus aureus* isolated from bovine mastitis. J. Anim. Sci..

[33] Duarte V.D., Treu L., Sartori C., Dias R.S., Paes I.D., Vieira M.S., Santana G.R., Marcondes M.I., Giacomini A., Corich V. (2020). Milk microbial composition of Brazilian dairy cows entering the dry period and genomic comparison between *Staphylococcus aureus* strains susceptible to the bacteriophage vB_SauM-UFV_DC4. Sci. Rep..

[34] García P., Madera C., Martínez B., Rodríguez A., Evaristo Suárez J. (2009). Prevalence of bacteriophages infecting *Staphylococcus aureus* in dairy samples and their potential as biocontrol agents. J. Dairy Sci..

[35] Geng H., Zou W., Zhang M., Xu L., Liu F., Li X., Wang L., Xu Y. (2020). Evaluation of phage therapy in the treatment of *Staphylococcus aureus*-induced mastitis in mice. Folia Microbiol.

[36] Hamza A., Perveen S., Abbas Z., Rehman S.U. (2016). The Lytic SA Phage Demonstrate Bactericidal Activity against Mastitis Causing *Staphylococcus aureus*. Open Life Sci..

[37] Iwano H., Inoue Y., Takasago T., Kobayashi H., Furusawa T., Taniguchi K., Fujiki J., Yokota H., Usui M., Tanji Y. (2018). Bacteriophage Phi SA012 Has a Broad Host Range against *Staphylococcus aureus* and Effective Lytic Capacity in a Mouse Mastitis Model. Biology.

[38] Synnott A.J., Kuang Y., Kurimoto M., Yamamichi K., Iwano H., Tanji Y. (2009). Isolation from sewage influent and characterization of novel *Staphylococcus aureus* bacteriophages with wide host ranges and potent lytic capabilities. Appl. Environ. Microbiol..

[39] Tanji Y., Tanaka A., Tani K., Kurimoto M., Miyanaga K. (2015). IgG-dependent aggregation of *Staphylococcus aureus* inhibits bacteriophage attack. Biochem. Eng. J..

[40] Jia H., Bai Q., Yang Y., Yao H. (2013). Complete Genome Sequence of *Staphylococcus aureus* Siphovirus Phage JS01. Genome Announc..

[41] Jia H., Dong W., Yuan L., Ma J., Bai Q., Pan Z., Lu C., Yao H. (2015). Characterization and complete genome sequence analysis of *Staphylococcus aureus* bacteriophage JS01. Virus Genes.

[42] Leite J.A., Pereira H.P., Borges C.A.V., Alves B.R.C., Ramos A., Martins M.F., Arcuri E.F. (2019). Lytic bacteriophages as a potential alternative to control *Staphylococcus aureus*. Pesqui. Agropecu. Bras..

[43] Li L.P., Zhang Z.Y. (2014). Isolation and characterization of a virulent bacteriophage SPW specific for *Staphylococcus aureus* isolated from bovine mastitis of lactating dairy cattle. Mol. Biol. Rep..

[44] Ngassam-Tchamba C., Duprez J.-N., Fergestad M., De Visscher A., L’Abee-Lund T., De Vliegher S., Wasteson Y., Touzain F., Blanchard Y., Lavigne R. (2020). In vitro and in vivo assessment of phage therapy against *Staphylococcus aureus* causing bovine mastitis. J. Glob. Antimicrob. Resist..

[45] Sangha K.K., Kumar B.V., Agrawal R.K., Deka D., Verma R. (2014). Proteomic Characterization of Lytic Bacteriophages of *Staphylococcus aureus* Isolated from Sewage Affluent of India. Int. Sch. Res. Not..

[46] Slanetz L.W., Jawetz E. (1941). Isolation and Characteristics of Bacteriophages for Staphylococci of Bovine Mastitis. J. Bacteriol..

[47] Song J., Ruan H., Chen L., Jin Y., Zheng J., Wu R., Sun D. (2021). Potential of bacteriophages as disinfectants to control of *Staphylococcus aureus* biofilms. BMC Microbiol..

[48] Srujana A.S., Sonalika J., Akhila D.S., Juliet M.R., Sheela P. (2022). Isolation of Phages and Study of their In vitro Efficacy on *Staphylococcus aureus* Isolates Originating from Bovine Subclinical Mastitis. Indian. J. Anim. Res..

[49] Teng F., Xiong X., Zhang S., Li G., Wang R., Zhang L., Wang X., Zhou H., Li J., Li Y. (2022). Efficacy Assessment of Phage Therapy in Treating *Staphylococcus aureus*-Induced Mastitis in Mice. Viruses.

[50] Wang Z.F., Zheng P.P., Ji W.H., Fu Q., Wang H.G., Yan Y.X., Sun J.H. (2016). SLPW: A Virulent Bacteriophage Targeting Methicillin-Resistant *Staphylococcus aureus* In vitro and In vivo. Front. Microbiol..

[51] Zhang L., Bao H., Wei C., Zhang H., Zhou Y., Wang R. (2015). Characterization and partial genomic analysis of a lytic Myoviridae bacteriophage against *Staphylococcus aureus* isolated from dairy cows with mastitis in Mid-east of China. Virus Genes.

[52] Zhang L.L., Sun L.C., Wei R.C., Gao Q., He T., Xu C.F., Liu X.J., Wang R. (2017). Intracellular *Staphylococcus aureus* Control by Virulent Bacteriophages within MAC-T Bovine Mammary Epithelial Cells. Antimicrob. Agents Chemother..

[53] Zhang Q., Xing S.Z., Sun Q., Pei G.Q., Cheng S., Liu Y.N., An X.P., Zhang X.L.L., Qu Y.G., Tong Y.G. (2017). Characterization and complete genome sequence analysis of a novel virulent Siphoviridae phage against *Staphylococcus aureus* isolated from bovine mastitis in Xinjiang, China. Virus Genes.

[54] Titze I., Krömker V. (2020). Antimicrobial Activity of a Phage Mixture and a Lactic Acid Bacterium against *Staphylococcus aureus* from Bovine Mastitis. Vet. Sci..

[55] Titze I., Lehnherr T., Lehnherr H., Krömker V. (2020). Efficacy of Bacteriophages Against *Staphylococcus aureus* Isolates from Bovine Mastitis. Pharmaceuticals.

[56] Bai Q., Yang Y., Lu C. (2016). Isolation and characterization of siphovirus phages infecting bovine *Streptococcus agalactiae*. Wei Sheng Wu Xue Bao.

[57] Gill J.J., Pacan J.C., Carson M.E., Leslie K.E., Griffiths M.W., Sabour P.M. (2006). Efficacy and pharmacokinetics of bacteriophage therapy in treatment of subclinical *Staphylococcus aureus* mastitis in lactating dairy cattle. Antimicrob. Agents Chemother..

[58] Gill J.J., Sabour P.M., Leslie K.E., Griffiths M.W. (2006). Bovine whey proteins inhibit the interaction of *Staphylococcus aureus* and bacteriophage K. J. Appl. Microbiol..

[59] O’Flaherty S., Coffey A., Meaney W.J., Fitzgerald G.F., Ross R.P. (2005). Inhibition of bacteriophage K proliferation on *Staphylococcus aureus* in raw bovine milk. Lett. Appl. Microbiol..

[60] Duarte V.D., Dias R.S., Kropinski A.M., Campanaro S., Treu L., Siqueira C., Vieira M.S., Paes I.D., Santana G.R., Martins F. (2018). Genomic analysis and immune response in a murine mastitis model of vB_EcoM-UFV13, a potential biocontrol agent for use in dairy cows. Sci. Rep..

[61] Guo M., Gao Y., Xue Y., Liu Y., Zeng X., Cheng Y., Ma J., Wang H., Sun J., Wang Z. (2021). Bacteriophage Cocktails Protect Dairy Cows Against Mastitis Caused By Drug Resistant *Escherichia coli* Infection. Front. Cell Infect. Microbiol..

[62] Porter J., Anderson J., Carter L., Donjacour E., Paros M. (2016). In vitro evaluation of a novel bacteriophage cocktail as a preventative for bovine coliform mastitis. J. Dairy Sci..

[63] Han G., Zhang J., Luo Z., Lu B., Zhang P., Yong K., Wang Y., Luo Y., Yang Z., Ren M. (2023). Characteristics of a novel temperate bacteriophage against Staphylococcus arlettae (vB_SarS_BM31). Int. Microbiol..

[64] Liang B., Zhao W., Han B., Barkema H.W., Niu Y.D., Liu Y., Kastelic J.P., Gao J. (2022). Biological and genomic characteristics of two bacteriophages isolated from sewage, using one multidrug-resistant and one non-multidrug-resistant strain of *Klebsiella pneumoniae*. Front. Microbiol..

[65] Shi Y., Zhao W., Liu G., Ali T., Chen P., Liu Y., Kastelic J.P., Han B., Gao J. (2021). Bacteriophages isolated from dairy farm mitigated *Klebsiella pneumoniae*-induced inflammation in bovine mammary epithelial cells cultured in vitro. BMC Vet. Res..

[66] Zhao W., Shi Y., Liu G., Yang J., Yi B., Liu Y., Kastelic J.P., Han B., Gao J. (2021). Bacteriophage has beneficial effects in a murine model of *Klebsiella pneumoniae* mastitis. J. Dairy Sci..

[67] Wang Z., Xue Y., Gao Y., Guo M., Liu Y., Zou X., Cheng Y., Ma J., Wang H., Sun J. (2021). Phage vB_PaeS-PAJD-1 Rescues Murine Mastitis Infected With Multidrug-Resistant Pseudomonas aeruginosa. Front. Cell Infect. Microbiol..

[68] Xi H.Y., He D.L., Li D., Liu S.S., Wang G., Ji Y.L., Wang X.W., Wang Z.J., Bi L.T., Zhao R.H. (2020). Bacteriophage Protects Against *Aerococcus viridans* Infection in a Murine Mastitis Model. Front. Vet. Sci..

[69] Zhang Q., Yu H., Sun Y., Zhangxiang L., Zhang P., Liu G., Qu Y., Tong Y., Li Y. (2017). Isolation and characterization of a lytic phage infecting Enterococcus faecium of bovine mastitis. Acta Vet. Zootech. Sin..

[70] Wu S., Fang Z., Tan J., Li M., Wang C., Guo Q., Xu C., Jiang X., Zhu H. (2021). DeePhage: Distinguishing virulent and temperate phage-derived sequences in metavirome data with a deep learning approach. Gigascience.

[71] Ferriol-González C., Domingo-Calap P. (2021). Phage Therapy in Livestock and Companion Animals. Antibiotics.

[72] Loc-Carrillo C., Abedon S.T. (2011). Pros and cons of phage therapy. Bacteriophage.

[73] Taslem Mourosi J., Awe A., Guo W., Batra H., Ganesh H., Wu X., Zhu J. (2022). Understanding Bacteriophage Tail Fiber Interaction with Host Surface Receptor: The Key “Blueprint” for Reprogramming Phage Host Range. Int. J. Mol. Sci..

[74] Kizziah J.L., Manning K.A., Dearborn A.D., Dokland T. (2020). Structure of the host cell recognition and penetration machinery of a *Staphylococcus aureus* bacteriophage. PLoS Pathog..

[75] Wang I.N., Smith D.L., Young R. (2000). Holins: The protein clocks of bacteriophage infections. Annu. Rev. Microbiol..

[76] Hendrix R.W., Smith M.C.M., Burns R.N., Ford M.E., Hatfull G.F. (1999). Evolutionary relationships among diverse bacteriophages and prophages: All the world’s a phage. Proc. Natl. Acad. Sci. USA.

[77] Mushegian A.R. (2020). Are There 10^31^ Virus Particles on Earth, or More, or Fewer?. J. Bacteriol..

[78] Clokie M.R., Millard A.D., Letarov A.V., Heaphy S. (2011). Phages in nature. Bacteriophage.

[79] Dedrick R.M., Guerrero-Bustamante C.A., Garlena R.A., Russell D.A., Ford K., Harris K., Gilmour K.C., Soothill J., Jacobs-Sera D., Schooley R.T. (2019). Engineered bacteriophages for treatment of a patient with a disseminated drug-resistant Mycobacterium abscessus. Nat. Med..

[80] Nale J.Y., Clokie M.R.J. (2021). Preclinical data and safety assessment of phage therapy in humans. Curr. Opin. Biotechnol..

[81] Principi N., Silvestri E., Esposito S. (2019). Advantages and Limitations of Bacteriophages for the Treatment of Bacterial Infections. Front. Pharmacol..

[82] Nale J.Y., Chutia M., Carr P., Hickenbotham P.T., Clokie M.R.J. (2016). ‘Get in Early’; Biofilm and Wax Moth (*Galleria mellonella*) Models Reveal New Insights into the Therapeutic Potential of Clostridium difficile Bacteriophages. Front. Microbiol..

[83] Malik D.J., Sokolov I.J., Vinner G.K., Mancuso F., Cinquerrui S., Vladisavljevic G.T., Clokie M.R.J., Garton N.J., Stapley A.G.F., Kirpichnikova A. (2017). Formulation, stabilisation and encapsulation of bacteriophage for phage therapy. Adv. Colloid. Interface Sci..

[84] Pedersen R.R., Krömker V., Bjarnsholt T., Dahl-Pedersen K., Buhl R., Jørgensen E. (2021). Biofilm Research in Bovine Mastitis. Front. Vet. Sci..

[85] Chen Y., Batra H., Dong J., Chen C., Rao V.B., Tao P. (2019). Genetic Engineering of Bacteriophages Against Infectious Diseases. Front. Microbiol..

[86] Gibb B., Hyman P., Schneider C.L. (2021). The Many Applications of Engineered Bacteriophages—An Overview. Pharmaceuticals.

[87] Guo D., Chen J., Zhao X., Luo Y., Jin M., Fan F., Park C., Yang X., Sun C., Yan J. (2021). Genetic and Chemical Engineering of Phages for Controlling Multidrug-Resistant Bacteria. Antibiotics.

[88] Suh G.A., Lodise T.P., Tamma P.D., Knisely J.M., Alexander J., Aslam S., Barton K.D., Bizzell E., Totten K.M.C., Campbell J.L. (2022). Considerations for the Use of Phage Therapy in Clinical Practice. Antimicrob. Agents Chemother..

[89] Vagenende V., Yap M.G., Trout B.L. (2009). Mechanisms of protein stabilization and prevention of protein aggregation by glycerol. Biochemistry.

[90] Rosner D., Clark J. (2021). Formulations for Bacteriophage Therapy and the Potential Uses of Immobilization. Pharmaceuticals.

